# Novel mechanism of miRNA‐365‐regulated trophoblast apoptosis in recurrent miscarriage

**DOI:** 10.1111/jcmm.13163

**Published:** 2017-04-10

**Authors:** Wei Zhao, Wei‐wei Shen, Xiao‐mei Cao, Wen‐yan Ding, Lin‐ping Yan, Ling‐juan Gao, Xiu‐Ling Li, Tian‐ying Zhong

**Affiliations:** ^1^ The Fourth School of Clinical Medicine of Nanjing Medical University Nanjing China; ^2^ State Key Laboratory of Reproductive Medicine Department of Clinical Laboratory, Obstetrics and Gynecology Hospital Affiliated to Nanjing Medical University, Tianfei Alley Nanjing China; ^3^ Dunman High School Singapore City Singapore

**Keywords:** Recurrent miscarriage, miRNA‐365, apoptosis, Glucocorticoid inducible kinase 1, mechanism

## Abstract

Clinical pregnancies increasingly end in recurrent miscarriage (RM) during the first trimester, with genetic factors shouldering the main responsibility. MicroRNAs (miRNAs) regulate gene expression in a wide array of important biological processes. We examined the potential role of dysregulated miRNAs in RM pathogenesis and trophoblast development as an approach to elucidate the molecular mechanism behind RM. miRNA profiles from clinical specimens of RM and induced abortion (IA) were compared, and several miRNAs were found to be aberrantly expressed in RM samples. Among the miRNAs, miR‐365 was significantly differentially expressed in RM decidual tissues. Furthermore, our results demonstrate that miR‐365 functions as an upstream regulator of MDM2/p53 expression, cell cycle progression and apoptosis in trophoblasts. Bioinformatic prediction and experimental validation assays identified SGK1 as a direct target of miR‐365; consistently, its protein levels were low in decidual tissues. Additionally, functional studies revealed that SGK1 silencing elicits cell cycle arrest and apoptosis in trophoblasts and that SGK1 overexpression attenuates the effects of miR‐365 on apoptosis and MDM2/p53 expression. Collectively, our data provide evidence that the up‐regulation of miR‐365 may contribute to RM by decreasing SGK1 expression, which suggests its potential utility as a prognostic biomarker and therapeutic target for RM.

## Introduction

RM, a common complication of early pregnancy, is defined as the occurrence of two or more pregnancy losses before 12 weeks’ gestation 1. The causal links of RM include several factors such as infectious 2, anatomic 3, endocrinologic 4, auimmunologic 5 and chromosomal 6. Despite substantial investigation concerning RM, in approximately 50% of cases, the detailed mechanisms still remain poorly characterized.

Pregnancy is an elaborate and complex process. During pregnancy, the proliferation and apoptosis of trophoblasts are closely regulated in a dynamic balance; if the balance is disturbed, apoptosis will predominate in the trophoblast cell growth process, resulting in abnormal placental development, aberrant foetal growth and adverse pregnancy outcomes 7, 8, 9. In 1998, Kokawa *et al*. first proposed that out‐of‐balance apoptosis in trophoblasts could be the main reason behind miscarriage 10. The transcription factor p53 regulates the cell cycle and apoptosis; and murine double minute 2 (MDM2), a downstream signalling molecule of p53, acts as a key negative regulator of p53 activity 11, 12. Studies suggest that MDM2 accelerates trophoblast growth, while p53 promotes apoptosis during trophoblast cellular development, and the balance between p53 and MDM2 is a key factor in the regulation of trophoblast apoptosis 13, 14. Therefore, the identification of factors that influence the expression or activity of p53 and MDM2 in trophoblasts is of potential importance in the development of targeted therapies for preventing RM.

miRNAs are single‐stranded RNA molecules of approximately 23 nucleotides that can regulate gene expression at the post‐transcriptional level through binding to the 3′‐untranslated regions (UTRs) of target genes 15, 16. Multiple reports have confirmed the role of miRNAs in the development of pathological pregnancy. Certain miRNAs, such as miR‐450a‐3p 17, repress cell proliferation in mouse embryonic fibroblast cells. Furthermore, three miRNA clusters (miR‐17, miR‐20a and miR‐20b) are dysregulated in placental tissues of women with severe eclampsia 18. In this study, we screened differentially expressed miRNAs in specimens from RM as compared to IA and identified miR‐365. Interestingly, miR‐365 has an established function in regulating the activity of the immune system and malignant tumours. For example, miR‐365 is reported to induce host immune defence by regulating IL‐6 expression 19. Moreover, miR‐365 is dysregulated in pancreatic cancer 20, endometriosis 21, lung cancer 22 and colon cancer 23, and it is involved in the development and progression of cutaneous squamous cell carcinoma 24. However, little has been reported regarding the relationship between miR‐365 and RM. Therefore, to explore the specific mechanism behind the miR‐365‐induced apoptosis of trophoblasts and the potential role of miR‐365 in the development and progression of RM, we sought to identify relevant downstream target genes of miR‐365. Our results demonstrate that miR‐365 targets SGK1 expression to modulate MDM2/p53 expression and enhance the apoptosis of trophoblasts, thus identifying a new pathway of regulation that may facilitate the prevention of RM.

## Materials and methods

### Cell lines

The human extravillous cytotrophoblast (EVCT)‐derived transformed cell lines HTR‐8/SVneo and HPT‐8 were obtained from Hangzhou Hibio Bio‐tech Co., Ltd (Hangzhou, Zhejiang, China). HTR‐8/SVneo cell lines were originally derived from HTR‐8, a short‐lived first‐trimester EVCT cell line that was immortalized with SV40 T antigen. HTR‐8/SVneo cells retain all of the functional and phenotypic characteristics of the parental HTR‐8 cells. Makers of EVCT include human placental lactogen, cytokeratins 18 and 8, human chorionic gonadotropin (hCG), type IV collagenase and human leucocyte antigen G (HLA‐G, a marker of extravillous trophoblasts) 25. HPT‐8, which is another immortalized primary cell clone, expresses cytokeratin 7, cytokeratin 18, vimentin, cluster of differentiation antigen 9, epidermal growth factor receptor, stromal cell‐derived factor 1 and placental alkaline phosphatase. HPT‐8 cells are positive for HLA‐G, secreted prolactin, estradiol, progesterone and hCG. These cells are permissive for the full replication cycle of human cytomegalovirus 26, 27.

### Participants and sampling

From February 2014 to January 2015, women who underwent IA or experienced RM at Nanjing Maternity and Child Health Care Hospital were included in this study. We collected information about the total number of previous pregnancies, the number of stillbirths and the number of previous miscarriages before collecting decidual tissues and blood samples. Relevant questionnaires were prepared for participants involved in this study by the Nanjing Maternity and Child Health Care Hospital.

Women (*n *=* *300) who had a previous history of RM confirmed by ultrasound scan were considered for the RM group. These women in the RM group had (*i*) an unexplained aetiology of RM with unexplained vaginal bleeding during 6–8 weeks of gestation that was not explained by verifiable factors known to be associated with RM, such as abnormal chromosomes, uterine abnormalities, hormonal and infection pathologies or immune disorders, or (*ii*) a history of two or more consecutive pregnancy losses in the first trimester. The average age was 28.8 years old, and the average gestational age was 56.4 days. The viabilities of the pregnancies were demonstrated by ultrasound scan by evaluating foetal heart activity a few days before RM. The women in the RM group were age‐matched with 300 women who had normal early pregnancies but underwent artificial abortion to terminate their unwanted pregnancies for family planning purposes (control group). The average age in the control group was 27.8 years, and the average gestational age was 56.6 days. Live pregnancies were confirmed by evaluating foetal heart activity by ultrasound on the day of termination. All of the women had regular menstrual cycles, and the gestational age estimates were based on the last menstrual period and confirmed by ultrasound. Termination of pregnancy was surgically achieved by vacuum suction, and the tissues and blood samples of participates were collected. Pregnancies with medical complications or diseases were excluded.

This study was performed in accordance with the ethical standards in the Declaration of Helsinki. The study was approved by the Ethical Committee of the Chinese Academy of Sciences and the Nanjing Maternity and Child Health Care Hospital in Nanjing (Number:201407; Date: 20 January, 2014). Written informed consent was obtained from all participants prior to sample collection.

### Tissue procurement and preparation

Decidual tissues were collected by suction curettage. Immediately after extraction of embryonic tissues of first‐trimester pregnancies from the uterus, decidual tissues were carefully dissected free of myometrial tissue or attached placenta and visible blood clots. After being washed twice in 0.9% NaCl, samples were stored at −80°C for further processing.

### miRNA microarray and clustering analysis

To compare miRNA expression between decidual tissues of RM (*n *=* *14) and IA samples (*n *=* *13), gene expression profiling was conducted using the PrimeView Human Gene Expression Array (Affymetrix), which contains 530,000 probes covering more than 36,000 transcripts and variants. Total RNA was hybridized according to the manufacturer's instructions. Six repeats were performed from each sample to ensure consistency of hybridization. All subsequent technical procedures and quality controls were performed by Genechem Co., Ltd., Shanghai, China. The arrays were scanned by a GeneChip Scanner 3000 (Affymetrix, inc., Santa Clara, CA, USA). GeneSpring GX software version 11.5 (Agilent Technologies, Palo Alto, CA, USA) was used to analyse the raw data obtained from each probe. Next, the data were normalized using the PLIER default protocol. Additionally, an unpaired *t*‐test was applied to analyse the significantly differentially expressed genes. Hierarchical clustering analysis was used to assess the relationship between significantly altered miRNAs in samples for each identified gene set with Euclidean distance and average linkage statistical methods.

### miR‐365‐lentivirus construction

The lentivirus gene transfer vector carrying the hsa‐miR‐365 precursor (Gene ID: 100126355) and encoding green fluorescence protein (GFP) was constructed by Genechem Co., Ltd., and confirmed by DNA sequencing. The primers were as follows: forward: GAT CTG CAG GGG TTA GCT TGG GGA CCT GAA C; reverse: GAT CAT ATG AGA GTG ACA TAC TGA TGC CTA C. The mutant 3′‐UTR was generated by the overlap‐extension PCR method. Both wild‐type and mutant 3′‐UTR fragments were subcloned into the pGL3‐control vector (Promega, Madison, WI, USA), downstream of the stop codon of the luciferase gene.

### Electron microscopy

Biopsies were taken from decidual tissues, and small blocks of tissues were obtained by cutting longitudinal sections of 3–5 mm maximum thickness. Next, the blocks were immersed immediately for 2 hrs in 2.5% glutaraldehyde. After being washed overnight in sodium phosphate buffer, the tissue blocks were post‐fixed in 1% OsO_4_ for 1 hr and stained with 1% uranyl acetate. Next, the tissue blocks were dehydrated and flat‐embedded in Durcupan (Fluka Chemic AG, Buchs, Switzerland) and sectioned to 60–70 nm thickness on 300 mesh copper slot grids. Finally, ultrathin sections were examined at 3700× and 12,500× magnification and photographs were taken using a Zeiss 109 electron microscope (Carl Zeiss, Oberkochen, Germany).

### HTR‐8/SVneo and HPT‐8 cell culture and DNA transfection

HTR‐8/SVneo and HPT‐8 cells were grown in Dulbecco's modified Eagle's medium/Ham's F‐12 medium supplemented with 1% nonessential amino acids, 2 mM glutamine and 10% heat‐inactivated foetal bovine serum in a 37°C incubator with 5% CO_2_. The wild‐type SGK1 cDNA (entire open reading frame including nucleotides 17–1436) was cloned using the RNA PCR Core Kit (Applied Biosystems, Foster City, CA, USA). The primers 5′‐GAC TGG ATC CTT CAC TGC TCC CCT CAG TCT TTT G‐3′ (sense) and 5′‐TAG CGT TAA CGG CAA CTC CAC CAA AGG CTA ACG AAA AC‐3′ (antisense) were used with the following cycling parameters: 94°C for 45 sec.; 60°C for 30 sec.; and 68°C for 80 sec. for 30 cycles, followed by 68°C for 20 sec. The PCR product was inserted in‐frame using BamHI/EcoRI sites of the pcDNA 3.1 vector. The resulting SGK1 vector was then transfected into HTR‐8/SVneo and HPT‐8 cells according to the manufacturer's protocol. Briefly, 750 μl of OptiMEM (Life Technologies, Gaithersburg, MD, USA) was used to dilute 500 pmol of SGK1 vector and 10 μl of Lipofectamine 2000. The solution was pre‐incubated for 45 min. at 37°C followed by incubation for an additional 15 min. at room temperature. Subsequently, the Lipofectamine 2000/SGK1 vector mixture was overlaid onto the cells. Finally, 1 ml of growth medium (20% foetal bovine serum) per well was added. Reporter gene activities were normalized to total protein levels. All of the results were from the average of triplicate experiments.

### Construction of an SGK1 shRNA expression plasmid

The SGK1 shRNA expression plasmid was constructed using the primers 5′‐CAG CUG AAA UGU ACG ACA A‐3′ (forward) and 5′‐UUC UCC GAA CGU GUC ACG U‐3′ (reverse) and pGenesil‐1 as the vector backbone. BamHI and HindIII restriction site overhangs were incorporated near the 5′ end of the two oligonucleotides; and a 6‐nucleotide poly‐T tract recognized as an RNA pol III termination signal was incorporated at the 3′ end of the shRNA template. The shRNA was synthesized, annealed and ligated into the BamHI and HindIII restriction sites of the pGenesil‐1 expression vector.

### Real‐time quantitative polymerase chain reaction (qPCR)

Total RNA was extracted from tissues using Trizol reagent (Invitrogen, Carlsbad, CA, USA) according to the manufacturer's instructions. The RNA was quantified by absorption at 260 nm. The isolated RNA was then DNase‐treated and reverse‐transcribed according to the manufacturer's recommended protocol. Briefly, miRNAs were reversely transcribed using the Primescript Reverse Transcription kit, miScript syBRGreen PCR kit and miScript Primer Assays according to the manufacturer's instructions (Qiagen, Valencia, CA. USA). Quantitative real‐time PCR was performed using an ABI PRISM 7300 sequence detection system. Cycling parameters were 2 min. at 50°C and 10 min. at 95°C, followed by a total of 40 cycles of 15 sec. at 95°C and 1 min. at 60°C. All of the reactions were performed in triplicate. The gene expression ▵▵CT values of miRNAs were calculated by normalizing to β‐actin as an internal control.

### Western blot analysis

HTR‐8/SVneo and HPT‐8 cells were collected in sample buffer and then incubated in lysis buffer and protease inhibitors for 30 min. on ice. Next, the supernatants were collected following centrifugation at 1.3 × 10^4^×*g* at 4°C for 15 min. Total proteins were electrophoresed through a 10–15% denaturing polyacrylamide gel and subsequently transferred to PVDF membranes. The membranes were then blocked for 1 hr in 5% non‐fat milk in PBS, 0.05% Tween 20, and the membranes were incubated at 4°C overnight with primary antibody. After incubation with the horseradish peroxidase‐conjugated secondary antibody for 1 hr at room temperature, relative protein bands intensities were quantified using the Enhanced Chemiluminescence Western Detection System.

### Detection of apoptotic cells

Apoptosis evaluation was performed by flow cytometric analysis using Annexin V‐FITC/propidium iodide (PI) staining. After experimental treatment, HTR‐8/SVneo and HPT‐8 cells were harvested, washed and resuspended in binding buffer composed of 10 mM HEPES, 140 mM NaCl and 2.5 mM CaCl_2_, pH 7.4. Then, the cells were incubated with Annexin V‐FITC and PI in the dark for 15 min. Finally, binding buffer was added, and the stained cells were analysed using a Beckman Coulter Epics XL flow cytometer. Q1_LL represents normal cells, and the early and the late apoptotic cells were located in the Q1_LR and Q1_UR regions. The necrotic cells were distributed in the Q1_UL region. The relative ratios of early and lately apoptotic cells were evaluated for comparison between samples.

### Cell cycle analysis

To determinate the cell cycle distribution, cells were washed with cold PBS at 48 hrs after transfection. Then, the cells were fixed in cold 70% ethanol overnight at −20°C. Subsequently, the cells were stained with PI (Sigma‐Aldrich, St. Louis, MO, USA) for 30 min. at room temperature. After staining, a FACS Calibur flow cytometer was used to evaluate the distribution of the cell cycle. The cell cycle fraction was determined using Modfit LT version 3.0 software (BD. Topsham, ME, USA).

### Immunohistochemical analysis

Immunohistochemistry for SGK1, MDM2 and p53 in decidual tissues was performed according to the manufacturer's instructions. Briefly, paraffin‐embedded sections of decidual tissues were deparaffinized in xylenes followed by rehydration in a series of graded alcohols (RT, 15 min.). The antigen retrieval was carried out in sodium citrate buffer (pH 6.0, 15 min.). Then, non‐specific binding sites were blocked by incubation with 5% normal goat serum (RT, 30 min.). The sections were incubated at 4°C overnight with primary antibodies against SGK1, MDM2 or p53. After rinsing with PBS buffer, the slides were incubated with secondary antibodies (30 min., 37°C) and 3, 3′‐diaminobenzidine (DAB) staining was applied to evaluate the chromogenic reaction.

### Luciferase assays

A mutant construct of SGK13′‐UTR or SGK3 3′‐UTR was obtained by introducing a mutation into the seven nucleotides (CCCGUAA) of the seed region for miR‐365. The miR‐365 target sequence in the coding region of SGK1or SGK3 was amplified by PCR and cloned into GV143 that contained a firefly luciferase reporter gene. Wild‐type SGK1/SGK3 3′‐UTR or mutant SGK1/SGK3 3′‐UTR and the empty 3′‐UTR vector were cotransfected into HEK293 cells, with Renilla luciferase vector transfection as reference. After incubation for 48 hrs, the cells were harvested and assayed for Renilla and firefly luciferase activities using the dual‐luciferase reporter assay system (Promega). The relative luciferase activities were calculated by normalizing to Renilla luciferase. Cells were transfected with empty 3′‐UTR vector as a negative control (NC).

### Statistical analysis

All experiments were repeated at least three times and performed in triplicate. Data are shown as means ± standard deviation (S.D.). *P*‐values less than 0.05 were considered to be statistically significant (*P <* *0.05; **P <* *0.01; ***P <* *0.001; ^#^ *P *>* *0.05). The Student's *t*‐test was used to assess differences between experimental groups.

## Results

### Identification of differentially expressed miRNAs in decidual tissue of RM and IA

**Figure 1:**
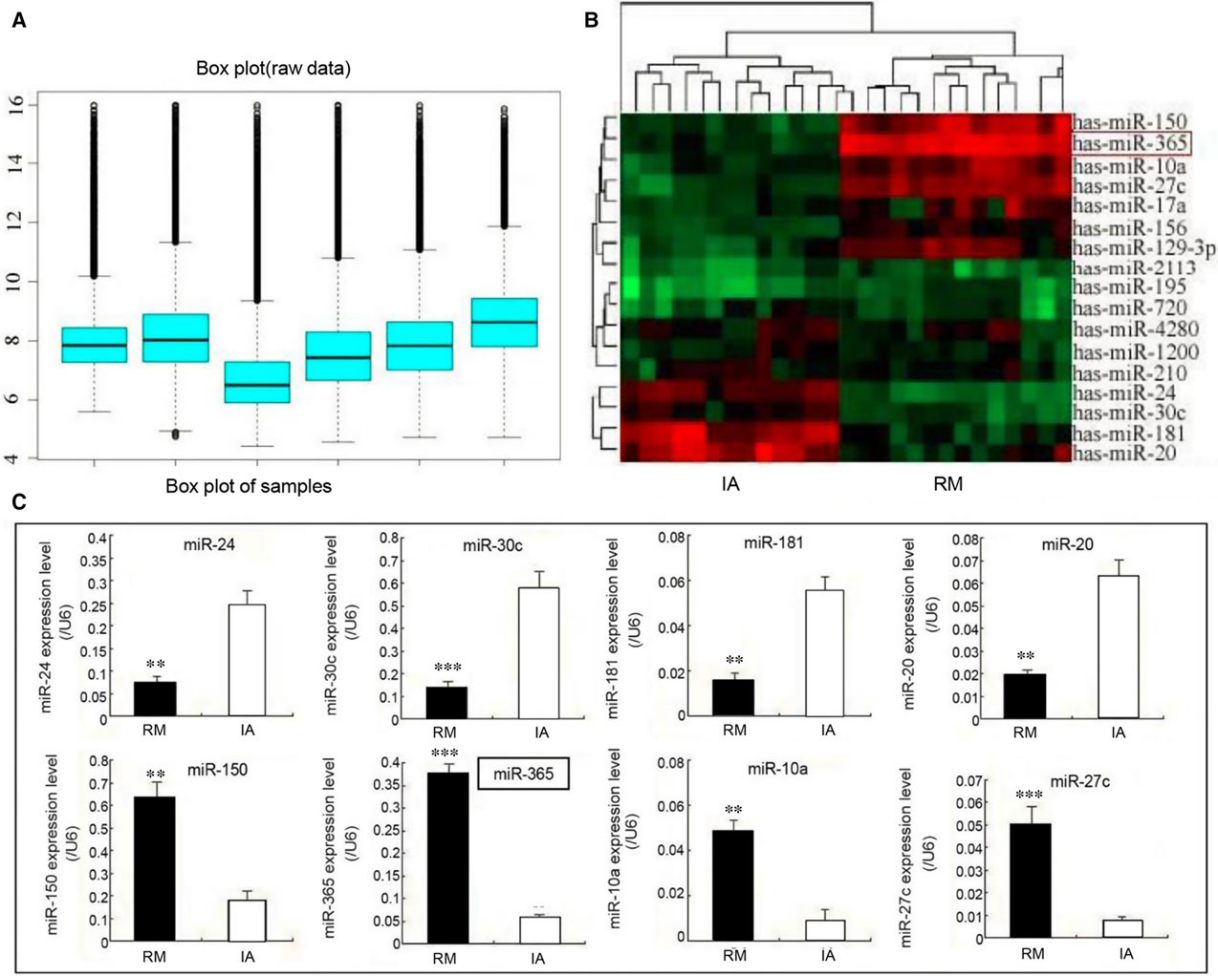
Identification of differentially expressed miRNAs in decidual tissues of RM and IA. (**A**) The distribution of signal values of the genes from microarray analysis was assessed using box line diagrams. The box plot analysis indicated that the distribution of the miRNA signal values was in good symmetrical shape. (**B**) Hierarchal clustering showing the profiling data of dysregulated miRNAs in RM tissues compared with IA tissues as determined by microarray analysis. Red indicates high relative expression, while green indicates low relative expression. (**C**) Differential expression of the four most highly up‐regulated miRNAs (miR‐150, miR‐365, miR‐10a, miR‐27c) and the four most highly down‐regulated miRNAs (miR‐24, miR‐30c, miR‐181, miR‐20) in decidual tissues of RM and IA was confirmed by qPCR. The data represent the means ± S.D. of three independent experiments (***P <* *0.001; **P <* *0.01).

To identify miRNAs that are dysregulated during RM, we assessed the profile of differentially expressed miRNAs for RM specimens *versus* IA specimens using microarray analysis (Fig. 1A). Hierarchical clustering showed a significant increase in the expression of seven miRNAs (miR‐150, miR‐365, miR‐10a, miR‐27c, miR‐17a, miR‐156, miR‐129‐3p) and a significant decrease in the expression of ten other miRNAs (miR‐20, miR‐181, miR‐30c, miR‐24, miR‐210, miR‐1200, miR‐4280, miR‐720, miR‐195, miR‐2113) (Fig. 1B). To verify the results of the microarray analysis, we assessed the expression of the four most highly up‐ and four most highly down‐regulated miRNAs by q‐PCR. The results confirm differential expression of each of these miRNAs (Fig. 1C).

### miR‐365 regulates MDM2 and p53 to induce apoptosis of trophoblasts

**Figure 2:**
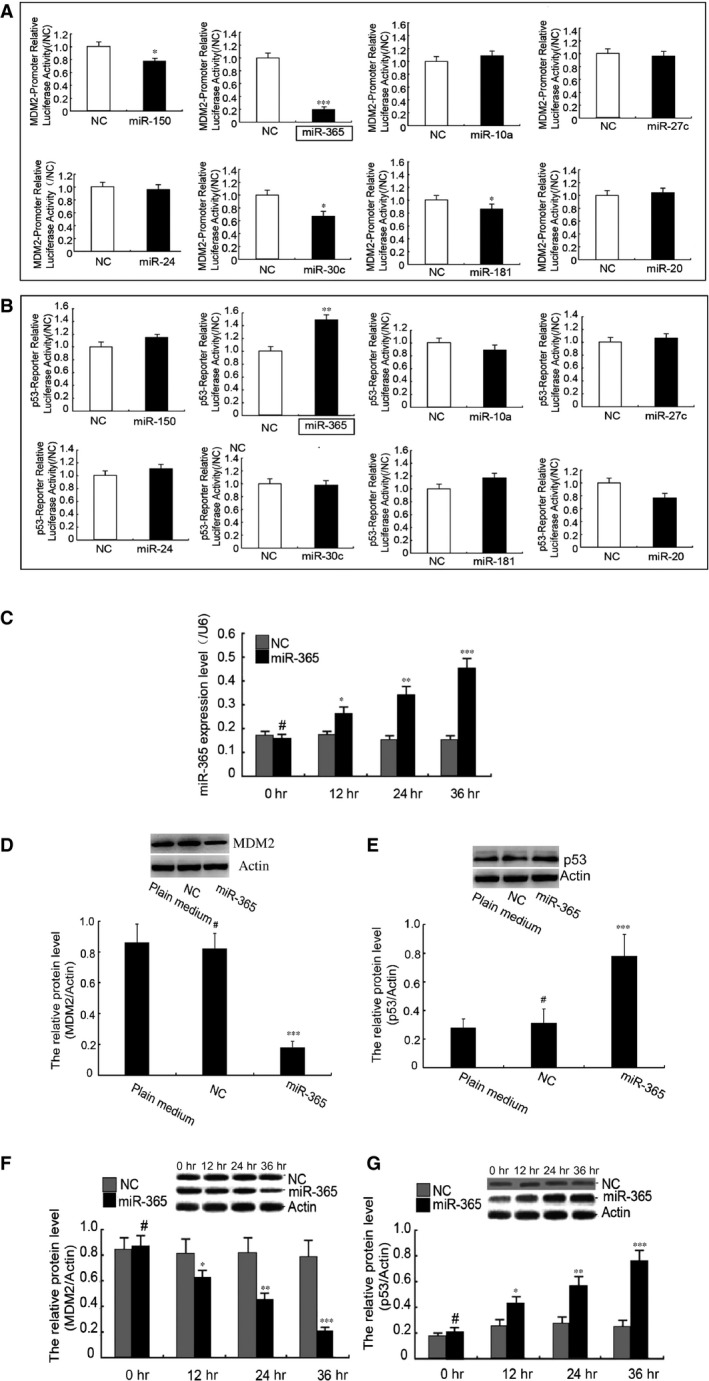
miR‐365 regulates MDM2 and p53 expression in trophoblasts. (**A** and **B**) Effects of the four most highly up‐regulated and down‐regulated miRNAs on MDM2 and p53 transcriptional activity. miRNA mimic or empty vector (NC) was cotransfected with the MDM2‐promoter‐reporter (panel A) or the p53‐reporter (panel A) into HEK293 cells. Renilla luciferase vector was transfected as reference. (**C**) Expression of miR‐365 after transfection with miR‐365 mimic in HTR 8/SVneo cells was verified by q‐PCR at different times post‐transfection. Similar results were found in HPT‐8 cells (data not shown). (**D** and **E**) At 36 transfection, the levels of MDM2 and p53 protein in the plain media group, the NC group and the miR‐365 group were measured using Western blot analysis. (**F** and **G**) The levels of MDM2 and p53 protein were assessed at different times after transfection. Expression was normalized to actin expression. The data represent means ± S.D. of three independent experiments. (***P <* *0.001; **P <* *0.01; *P <* *0.05; ^#^ *P *>* *0.05 *versus* NC). Similar results were found in HPT‐8 cells (data not shown).

We reasoned that miRNAs that have functional relevance to RM may be involved in the control of apoptosis in trophoblasts. Furthermore, our initial results demonstrated that MDM2 is expressed at reduced levels in RM relative to IA tissues (Fig. S1). MDM2 is a negative regulator of the tumour suppressor gene p53, which is known to promote apoptosis during trophoblast cellular development^.^ Therefore, we prepared promoter/reporter constructs for p53 and MDM2 and cotransfected each promoter/reporter construct with each of the eight most highly up‐ and down‐regulated miRNAs. As shown in Figure 2A and B, the MDM2 reporter activity was significantly decreased, while the p53 reporter activity was significantly increased by miR‐365. miR‐30c and miR‐181 also suppressed MDM2 promoter activity, but there were no other apparent changes observed in the reporter activities by the other miRNAs. Based on these data, we hypothesized that miR‐365 may regulate MDM2/p53 to induce apoptosis of trophoblasts.

To verify that miR‐365 functions as an upstream regulator of MDM2 and p53, we transfected miR‐365 mimic into the human EVCT‐derived transformed cell lines HTR 8/Svneo and HPT‐8. qPCR analysis confirmed that miR‐365 was expressed in trophoblast cells after transfection in a time‐dependent manner (Fig. 2C). Furthermore, 36 hrs after transfection, the level of MDM2 protein was significantly decreased, while the level of p53 protein levels was obviously increased (Fig. 2D and E). The decrease in MDM2 and the increase in p53 were in a time‐dependent manner (Fig. 2F and G).

**Figure 3:**
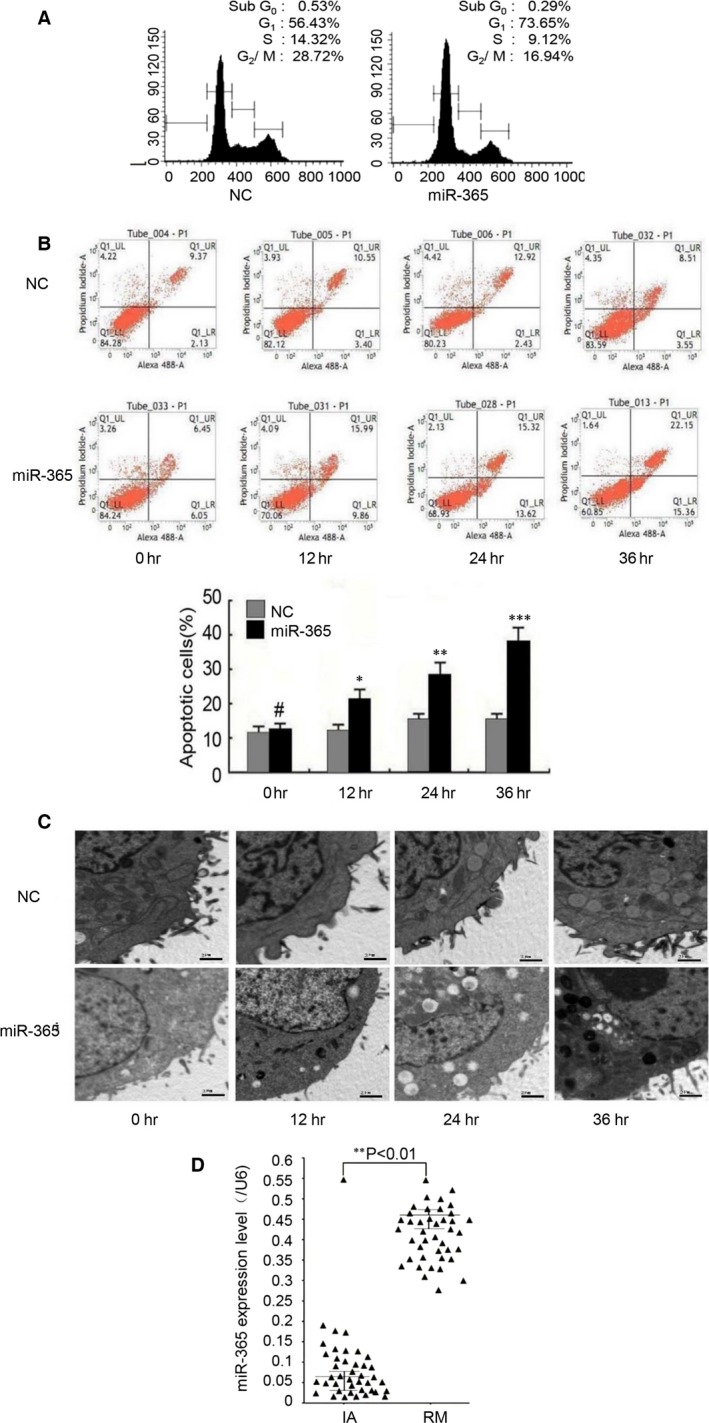
Effects of miR‐365 overexpression on the cell cycle distribution and apoptosis of trophoblast cells. (**A**) The cell cycle distribution was assessed by flow cytometry with PI staining in HTR 8/SVneo trophoblast cells after transfection with miR‐365 mimic or empty vector (NC). The percentages of cells in G1, S and G2 cell cycle phases are shown. Similar results were found in HPT‐8 cells (data not shown). (**B**) HTR 8/SVneo cells were stained with annexin V and PI followed by analysis by flow cytometry. The data represent as the means ± S.D. of three independent experiments (***P <* *0.001; **P <* *0.01; *P <* *0.05; ^#^ *P *>* *0.05 *versus* NC). Similar results were found in HPT‐8 cells (data not shown). (**C**) Electron microscopy images showing typical morphologic changes in trophoblast cells, including nuclear chromatin condensation and formation of apoptotic bodies in HTR 8/SVneo cells after transfection with NC or miR‐365 mimic. Scale bar, 2 μm. Similar results were found in HPT‐8 cells (data not shown). (**D**) The miR‐365 expression levels in 40 pairs of RM decidual tissues compared with 40 matched IA samples were assessed by q‐PCR (**P <* *0.01, calculated using the pairwise *t*‐test).

To evaluate the functional consequence of the dysregulation of apoptosis‐related genes by miR‐365, we assessed the effects of miR‐365 on trophoblast cell growth and apoptosis by flow cytometry. At 36 hrs after transfection, miR‐365 caused an obvious cell cycle arrest at the G1 phase (Fig. 3A). Furthermore, overexpression of miR‐365 induced HTR 8/Svneo and HPT‐8 cell apoptosis in a time‐dependent manner (Fig. 3B). As verification, the miR‐365‐induced apoptosis was inhibited by anti‐miR‐365 (Fig. S2). To further confirm these findings, we evaluated the ultrastructural changes in trophoblast cells at different time points by transmission electron microscopy. Apoptosis was obvious in miR‐365‐transfected HTR 8/Svneo and HPT‐8 cells by 12 hrs post‐transfection; at 24 and 36 hrs, the nuclear chromatin appeared condensed and apoptotic bodies were apparent (Fig. 3C). These findings confirm that miR‐365 induces apoptosis in trophoblasts, which is likely to be a consequence of its regulation of MDM2 and p53.

To verify the dysregulation of miR‐365 in RM, we obtained 40 IA and 40 RM samples and evaluated the expression in each of the samples by qPCR. The results show that miR‐365 was up‐regulated in RM by 6.17‐fold, which is similar to the 6.51‐fold up‐regulation observed by microarray analysis for this miRNA (Fig. 3D). Therefore, these findings support a model in which up‐regulated miR‐365 expression under conditions of RM leads to a decrease in MDM2, an increase in p53, cell cycle arrest and apoptosis of trophoblasts.

### Identification of SGK1 as a target of miR‐365 that is down‐regulated in RM

**Figure 4:**
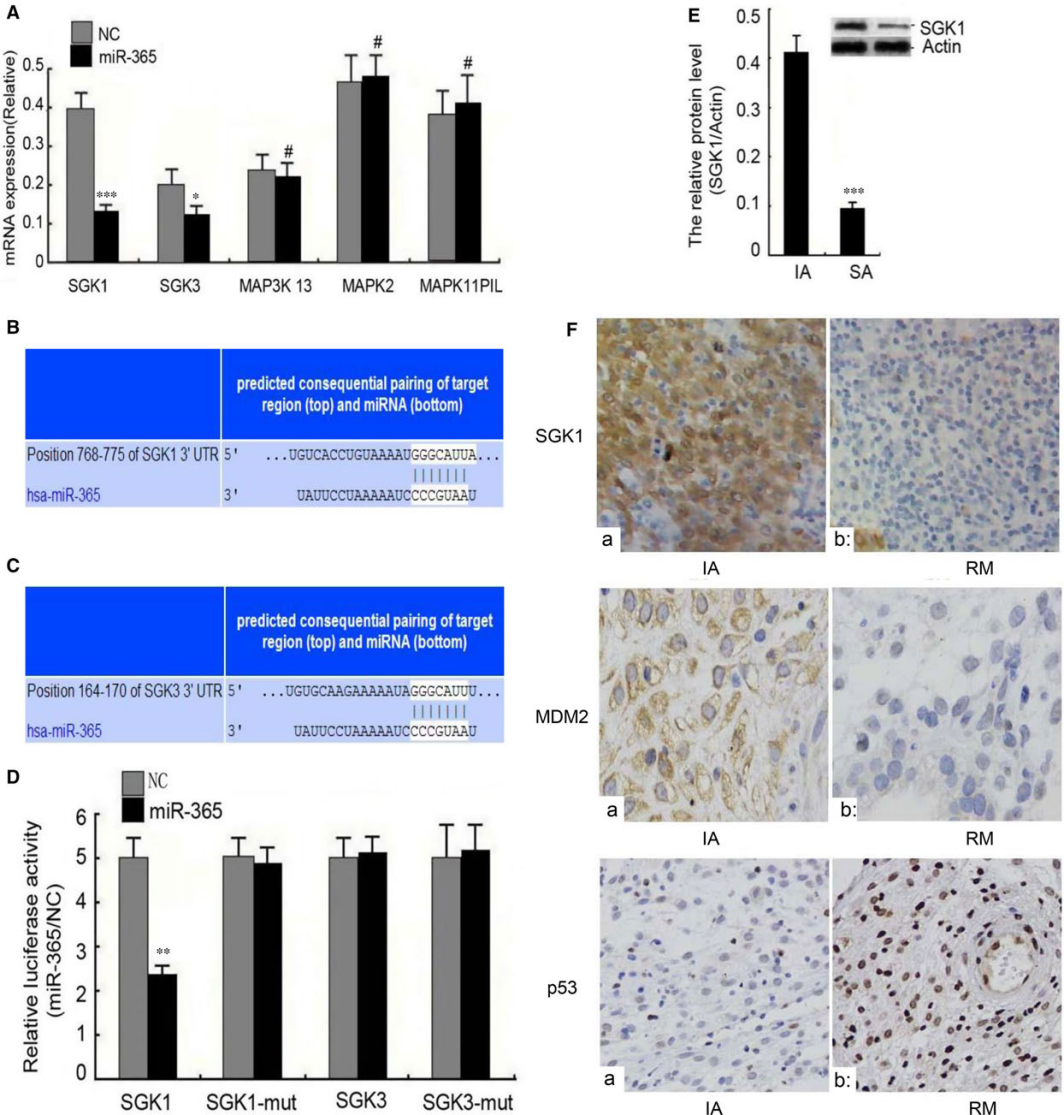
Identification of SGK1 as an miR‐365 target gene that is dysregulated in RM. (**A**) HTR 8/SVneo cells were treated with miR‐365 mimic or empty vector (NC). At 36 hrs post‐transfection, the mRNA expression levels of genes in the SGK and MAPK pathway were analysed by q‐PCR. Similar results were found in HPT‐8 cells (data not shown). (**B** and **C**) Putative target sites in the 3′ UTRs of SGK1 (Position 768–775) and SGK3 (Position 164–170). 3′ UTR complementary to the miR‐365 sequence were predicted by target scan. (**D**) SGK1, SGK1 mut, SGK3 and SGK3 mut 3′‐UTR luciferase reporters were cotransfected into HEK293 cells with miR‐365 mimic or empty vector (NC). At 36 hrs post‐transfection, the luciferase activity was assessed using the dual‐luciferase reporter assay system. The relative luciferase activity was normalized to Renilla luciferase activity. (**E**) The relative expression level of SGK1 protein in RM relative to IA was evaluated by Western blotting. Actin protein was assessed as a loading control. The data represent the means ± S.D. of three independent experiments (***P <* *0.001; **P <* *0.01; *P <* *0.05; ^#^ *P *>* *0.05 *versus* NC). (**F**) The relative expression levels of SGK1, MDM2 and p53 were assessed by immunohistochemistry in decidual tissues from IA tissues (a) and RM tissues (b). Original magnification 200×.

To identify additional genes and pathways regulated by miR‐365, including potential genes that are directly regulated by miR‐365, we performed bioinformatic analysis (www.targetscan.org). SGK and mitogen‐activated protein kinase (MAPK) signalling pathways had the highest relevance to miR‐365 (*P *<* *0.001), while phosphoinositide3‐kinase/Akt (PI3‐k/Akt) and the nuclear receptor subfamily were also predicted to comprise potential target pathways of miR‐365 (Table 1). As a result, SGK and MAPK were selected for further evaluation as potential miR‐365 targets. Transfection of miR‐365 did not significantly affect the mRNA levels of MAP3K13, MAPK2 or MAPK11P1L; however, miR‐365 significantly decreased the mRNA levels of both SGK1 and SGK3 (Fig. 4A). Additionally, SGK1 (position 768–775) and SGK3 (position 164–170) each has a site in the 3′‐UTR that is a perfect match to the miR‐365 seed sequence CCCGUAA (Fig. 4B and C). Furthermore, both sites are evolutionarily conserved among multiple species with homology reaching up to 100% (Fig. S3A and B).

**Table 1:** miR‐365 target genes biological information analysis

Pathway name	Genes	*P* value
SGK signalling pathway	SGK1; SGK3	<0.0001
MAPK signalling pathway	MA3K13; MAPK2; MAPK11P1L	<0.0001
Nuclear receptor subfamily	NR1D2; NR3C2; NR2C2; NR3C1	0.0004
PI3‐k/Akt signalling pathway	PIK3R3; AKT3; SGK1; SGK3	0.05

To further evaluate the potential function of SGK1 and SGK3 as targets of miR‐365, we constructed luciferase reporter plasmids containing SGK1 or SGK3 wild‐type or mutant 3′‐UTR sequences. Our results demonstrate that the activity of the SGK1 reporter, but not the SGK1‐mut reporter, was reduced specifically by cotransfection of miR‐365. The SGK3 reporter was not affected by miR‐365 cotransfection (Fig. 4D). These results suggest that miR‐365 directly targets the 3′‐UTR sequence of SGK1, but not SGK3. The ability of miR‐365 to specifically target SGK1 was confirmed by Western blotting (Fig. S4A and B).

To determine whether SGK1 is differentially expressed in decidual tissues from RM as compared to IA, we performed Western blotting. The results demonstrate a significant reduction in SGK1 levels in RM patients *versus* IA patients (Fig. 4E). Furthermore, immunohistochemical analysis confirmed that SGK1 and MDM2 are underexpressed in the same RM tissues in which p53 is overexpressed (Fig. 4F). These results are consistent with the possibility that SGK1 functions as a direct target of miR‐365 in RM.

### SGK1 regulates apoptosis in trophoblasts through a mechanism involving miR‐365, MDM2 and p53

**Figure 5:**
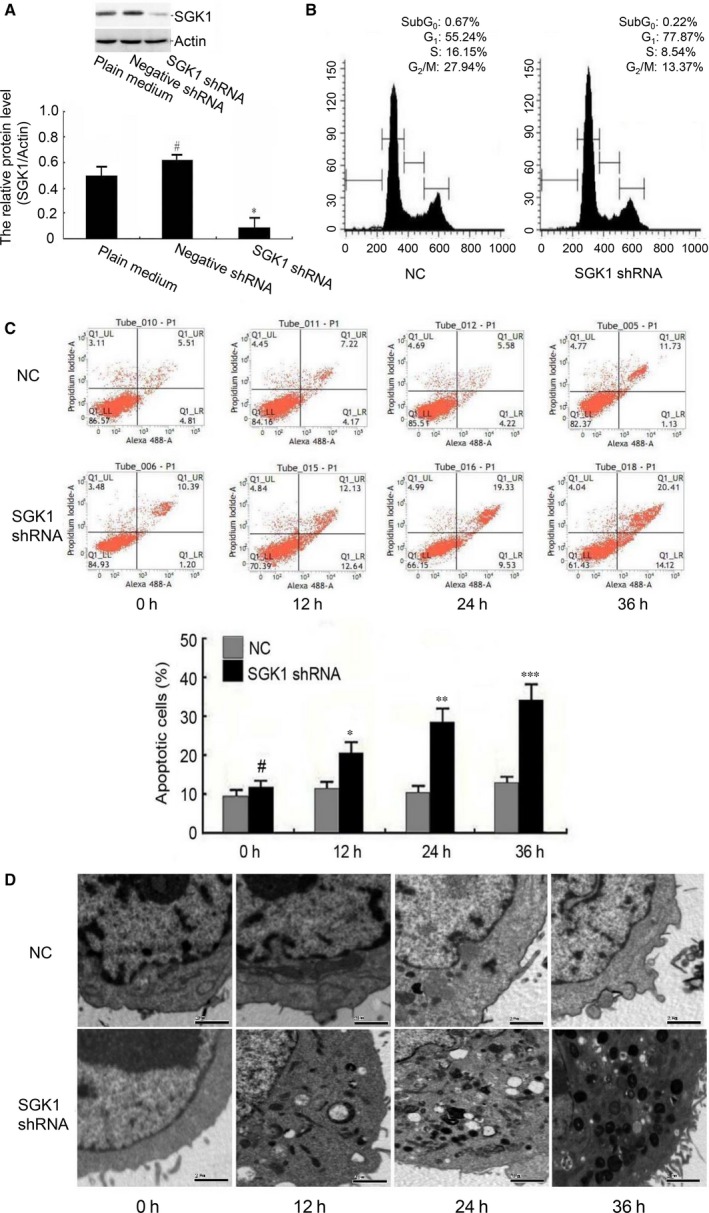
Effects of SGK1 on trophoblast cell apoptosis. (**A**) HTR 8/SVneo cells were transfected with SGK1 shRNA and empty vector (NC). After transfection, the expression levels of SGK1 after transfection with SGK1 shRNA in HTR 8/SVneo cells were verified by Western blotting. Expression was normalized to actin. The data represent the means ± S.D. of three independent experiments (*P <* *0.05; ^#^ *P *>* *0.05 *versus* plain medium). Similar results were found in HPT‐8 cells (data not shown). (**B**) After 36 hrs, the cell cycle distribution was detected by PI staining and flow cytometry. The percentages of cells in G1, S and G2 cell cycle phases are shown. Similar results were found in HPT‐8 cells (data not shown). (**C**) HTR 8/SVneo cells were transfected with SGK1 shRNA and empty vector (NC). At different times post‐transfection, cells were stained with annexin V and followed by analysis by flow cytometry. The data represent the means ± S.D. of three independent experiments (***P <* *0.001; **P <* *0.01; *P <* *0.05; ^#^ *P *>* *0.05 *versus* NC). Similar results were found in HPT‐8 cells (data not shown). (**D**) HTR 8/SVneo cells were treated with SGK1 shRNA or empty vector (NC). At different times, the ultrastructure of the cells was examined by transmission electron microscopy. Scale bar, 2 μm. Similar results were found in HPT‐8 cells (data not shown).

To further elaborate the potential role of SGK1 in apoptosis of trophoblasts in RM, we used shRNA to reduce SGK1 expression by lentivirus‐mediated RNA interference technology (Fig. 5A). Flow cytometric apoptosis assay showed that SGK1 knockdown resulted in a time‐dependent cell cycle arrest at the G1 phase in HTR 8/Svneo and HPT‐8 trophoblasts (Fig. 5B). Furthermore, SGK1 knockdown increased the level of apoptosis, which was detected both by flow cytometry (Fig. 5C) and transmission electron microscopy (Fig. 5D). Collectively, these results demonstrate that the effects of SGK1 knockdown are similar to the effects of miR‐365 accumulation, which further supports the possibility that SGK1 acts as a direct functional target of miR‐365.

**Figure 6:**
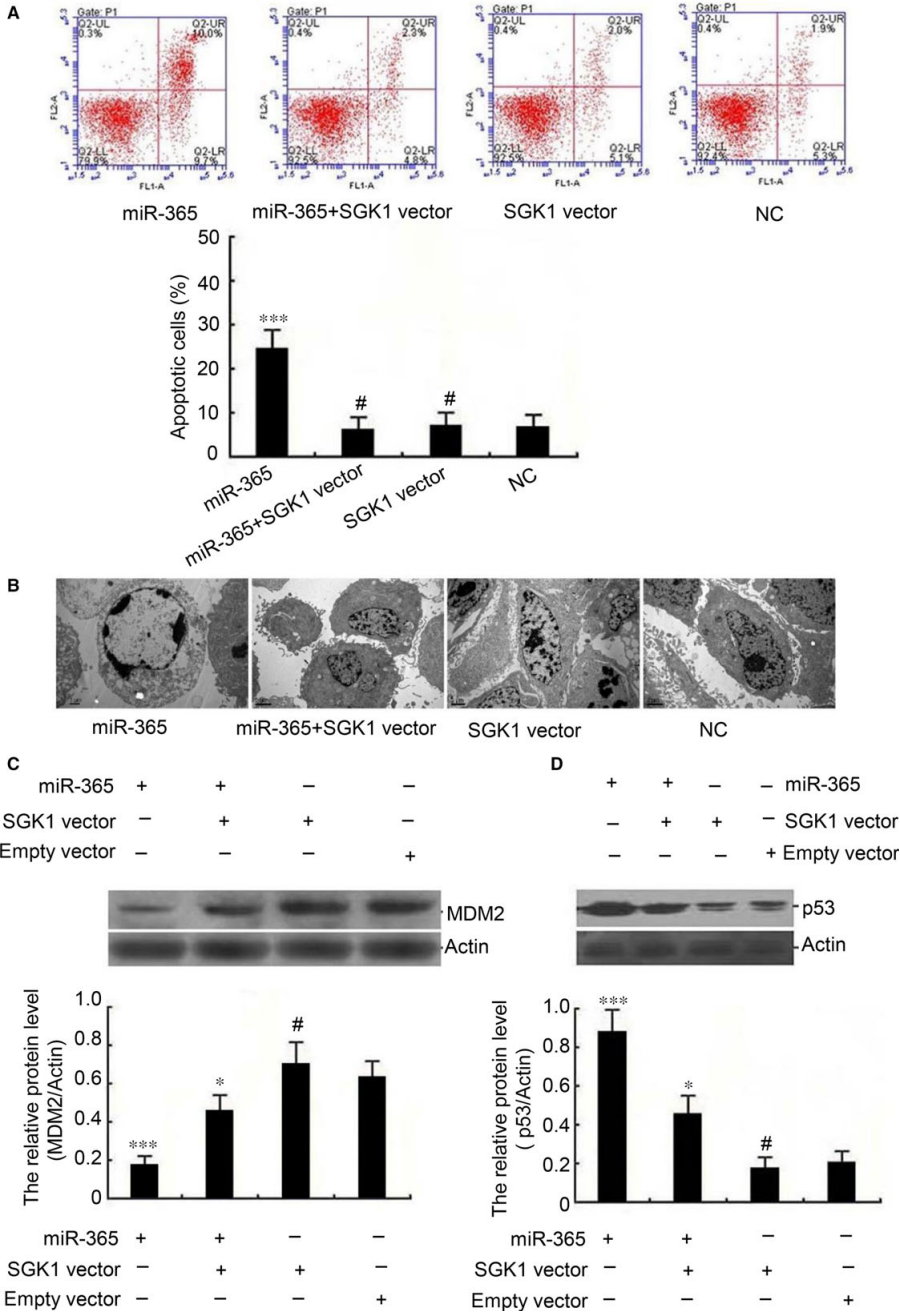
SGK1 overexpression counteracts the miR‐365 regulated‐signalling pathway towards apoptosis in trophoblasts. HTR 8/SVneo cells were treated with miR‐365, miR‐365 + SGK1 vector, SGK1 vector or empty vector (NC). Similar results were found in HPT‐8 cells (data not shown). (**A**) At 36 hrs post‐transfection, the cells were stained with annexin V and analysed by flow cytometry. Similar results were found in HPT‐8 cells (data not shown). (**B**) Transmission electron microscopy of the cells from panel A. Scale bar, 2 μm. Similar results were found in HPT‐8 cells (data not shown). (**C** and **D**) The levels of MDM2 and p53 protein were measured by Western blot analysis after transfection as in panel A. Relative MDM2 and p53 protein levels were normalized to the levels of actin. The data represent the means ± S.D. of three independent experiments. (p* < 0.05; ***P <* *0.001; ^#^ *P *>* *0.05 *versus* NC). Similar results were found in HPT‐8 cells (data not shown).

To verify the role of SGK1 in mediating apoptosis downstream of miR‐365 in trophoblasts, we assessed whether SGK1 overexpression can reverse the effects of miR‐365 on apoptosis. Flow cytometric apoptosis assay revealed that SGK1 vector alone had no effect on apoptosis; however, SGK1 vector attenuated the effects of miR‐365 mimic in promoting apoptosis (Fig. 6A). The results were verified by transmission electron microscopy (Fig. 6B).

Given the roles of MDM2 and p53 in the process of trophoblast cellular apoptosis (Fig. 3), as well as their demonstrated dysregulation in RM (Fig. 4F), we evaluated whether SGK1 expression might counteract the miR‐365‐mediated effects on MDM2 and p53 expression. SGK1 expression alone had no obvious effect on the expression of either MDM2 or p53; however, SGK1 attenuated the effects of miR‐365 mimic both in decreasing MDM2 expression and in enhancing p53 expression (Fig. 6C and D). These data suggest that miR‐365‐induced apoptosis of trophoblasts may be mediated by SGK1 signalling in the progression towards RM.

## Discussion

When placentas in the maternal–foetal interface suffer the composite actions of various factors, the trophoblasts are confronted with abnormal proliferation, differentiation, invasion and apoptosis, which provide a cytological basis for RM 27, 28, 29, 30. Previous studies have mainly revolved around the invasion of trophoblasts into endometrial stroma and the reshaping of blood vessels as mechanisms behind RM. Recently, increasing evidence has shown excessive apoptosis of trophoblasts, which not only results in the destruction of cellular structural integrity, but also aggravates or leads to the dysfunction of trophoblasts, which suggests that trophoblasts apoptosis is a pivotal event in RM development 31, 32. Consequently, further study is important to characterize the essential properties of trophoblasts and explore the key factors regulating the apoptosis of trophoblasts.

Existing investigations have uncovered the molecular mechanism of trophoblasts apoptosis at the genetic level, but the efficacy of genetic therapies is not remarkable 33. In recent years, miRNAs, which are small non‐coding RNA molecules, have been a widespread focus of research due to their roles in the development of diseases. Notably, because of the sequence‐specific interaction of miRNAs with one or more target genes, miRNAs have been explored as key regulatory molecules with potential therapeutic capabilities in refractory and complex diseases. The association between miRNA expression and pathological pregnancy has been reported 34. However, little is known about the relationship between miRNAs and RM. Therefore, we obtained decidual tissues from RM and IA and performed microarray to identify differentially expressed miRNAs. We identified seven up‐regulated miRNAs and ten down‐regulated miRNAs, and among the miRNAs, miRNA‐365 was unique in that it could repress the activity of MDM2 and enhance the activity of p53. Therefore, we speculated that miR‐365 might be an upstream trophoblastic apoptosis‐related regulator of MDM2/p53 with an integral role in the occurrence of RM.

Previous studies have demonstrated that miR‐365 is dysregulated to varying degrees in some human diseases such pancreatic cancer 20 and endometriosis 21, in which it is up‐regulated, and lung cancer 22 and colon cancer 23, in which it is down‐regulated. Furthermore, in these studies, miR‐365 is shown to exert its tumour‐suppression or tumour‐promotion function by targeting apoptosis‐relevant genes. For example, miR‐365 induces gemcitabine resistance in pancreatic cancer cells by targeting the adaptor protein SHC1 and the pro‐apoptotic regulator BAX 20. Furthermore, miRNA‐365 inhibits cell cycle progression and promotes the apoptosis of colon cancer cells by targeting Cyclin D1 and Bcl‐2 23. We demonstrated that overexpression of miR‐365 in trophoblast cells can induce cell cycle arrest at the G1 phase and cellular apoptosis, suggesting that miR‐365 acts as an apoptotic regulator in the development of RM.

To assess the mechanism behind miR‐365‐induced apoptosis of trophotosis, we applied bioinformatic analysis to predict downstream target genes of miR‐365. Four molecular pathways (SGK, MAPK, PI3‐k/Akt signalling pathway and nuclear receptor subfamily) were predicted to be regulated by miR‐365. Among them, SGK and MAPK signalling pathways had the highest potential relevance to miR‐365 (*P *<* *0.001). Furthermore, we determined that miR‐365 expression suppresses both SGK 1 and SGK 3 expression, but that it has no effect on the expression of the MAPK signalling pathway genes MAP3K13, MAPK2 and MAPK11P1L. Therefore, we focused our efforts on the SGK pathway. Further experimentation demonstrated that a reporter construct bearing the 3′‐UTR of SGK1, but not SGK3, was repressed by miR‐365 mimic. Based on these findings, we proposed SGK1 as a direct target gene of miR‐365 in RM.

As members of kinase subfamily, both SGK1 and SGK3 are regulated primarily at the transcriptional level. They act as potent apoptosis suppressors in response to a variety of extracellular stimuli 35, 36, 37. As a key subtype of SGK family, SGK1 has been reported its abnormal expression in cycling endometrium interferes with embryo implantation, leading to infertility, or predisposes to pregnancy complications 38. Our data demonstrate the significantly down‐regulated expression of SGK1 in decidual tissues and the loss of SGK1 can obviously influence trophoblasts growth and apoptosis. Consistently, Wang *et al*. 39 have reported low levels of SGK1 expression in patients with miscarriages and its inverse correlation with miR‐199b‐5p. This suggests that there are likely to be other miRNAs that are involved in the down‐regulation of SGK1. Previous studies have demonstrated that SGK1 regulates cell survival, proliferation and differentiation through MDM2 or p53 40, 41. Interestingly, in our study, overexpression of miR‐365 resulted in corresponding changes in p53 and MDM2 protein levels. Consequently, it is possible that miR‐365‐induced apoptosis involves the SGK1‐mediated alterations in p53 and MDM2 protein levels.

Collectively, our findings demonstrate the up‐regulation of miR‐365 in tissues from patients with RM, with SGK1 as a direct target of miR‐365 that regulates trophoblast apoptosis. Further investigation may help to determine whether miR‐365 may also repress SGK1, leading to MDM2/p53‐mediated apoptosis and cell cycle arrest of trophoblasts in ectopic pregnancies and other types of pathological pregnancy. Given these findings, the interaction between miR‐365 and its target gene SGK1 could potentially be explored as a prognostic indicator or therapeutic target to improve clinical treatment of poor pregnancy outcomes such as RM.

## Conflict of interest

The authors confirm that there is no conflict of interest.

## Supporting information


**Figure S1** Reduced MDM2 expression in RM.

**Figure S2** HTR 8/SVneocells were treated with miR‐365 (group1), miR‐365 + anti‐ miR‐365 (group2), or empty vector (NC, group3).

**Figure S3** The putative miR‐365 target sequences in the 3′ UTRs of SGK1 and SGK3 are highly conserved.

**Figure S4** SGK1 is a target of miR‐365.
