# Supplementary figures and images for: Novel mechanism of miRNA‐365‐regulated trophoblast apoptosis in recurrent miscarriage
Source: J Cell Mol Med. 2017 Apr 10;21(10):2412–25. doi: 10.1111/jcmm.13163 (PMC5618703; doi:10.1111/jcmm.13163)

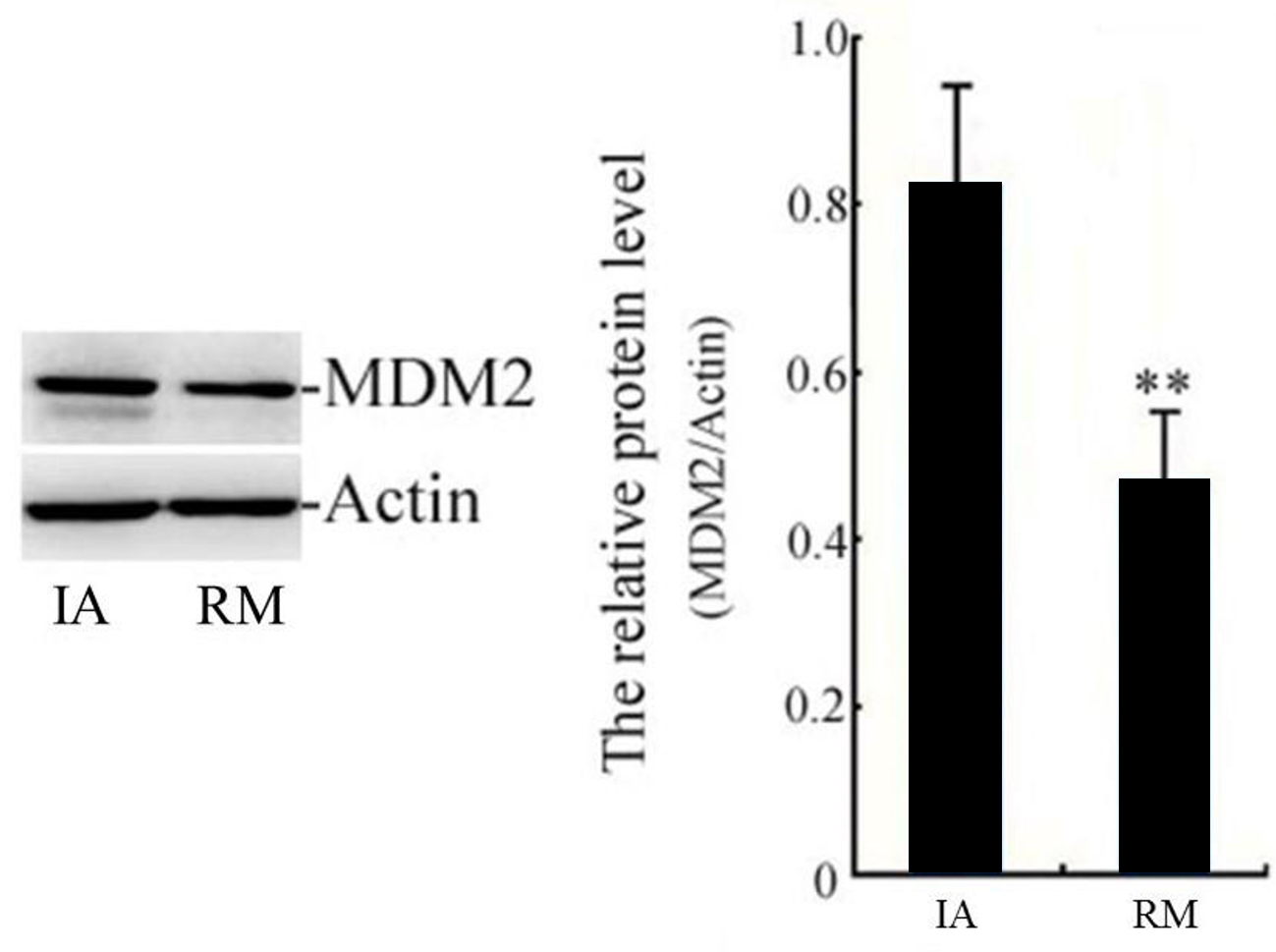

Supplement: Supplementary file 1 — Figure S1 Reduced MDM2 expression in RM. [file JCMM-21-2412-s001.tif]

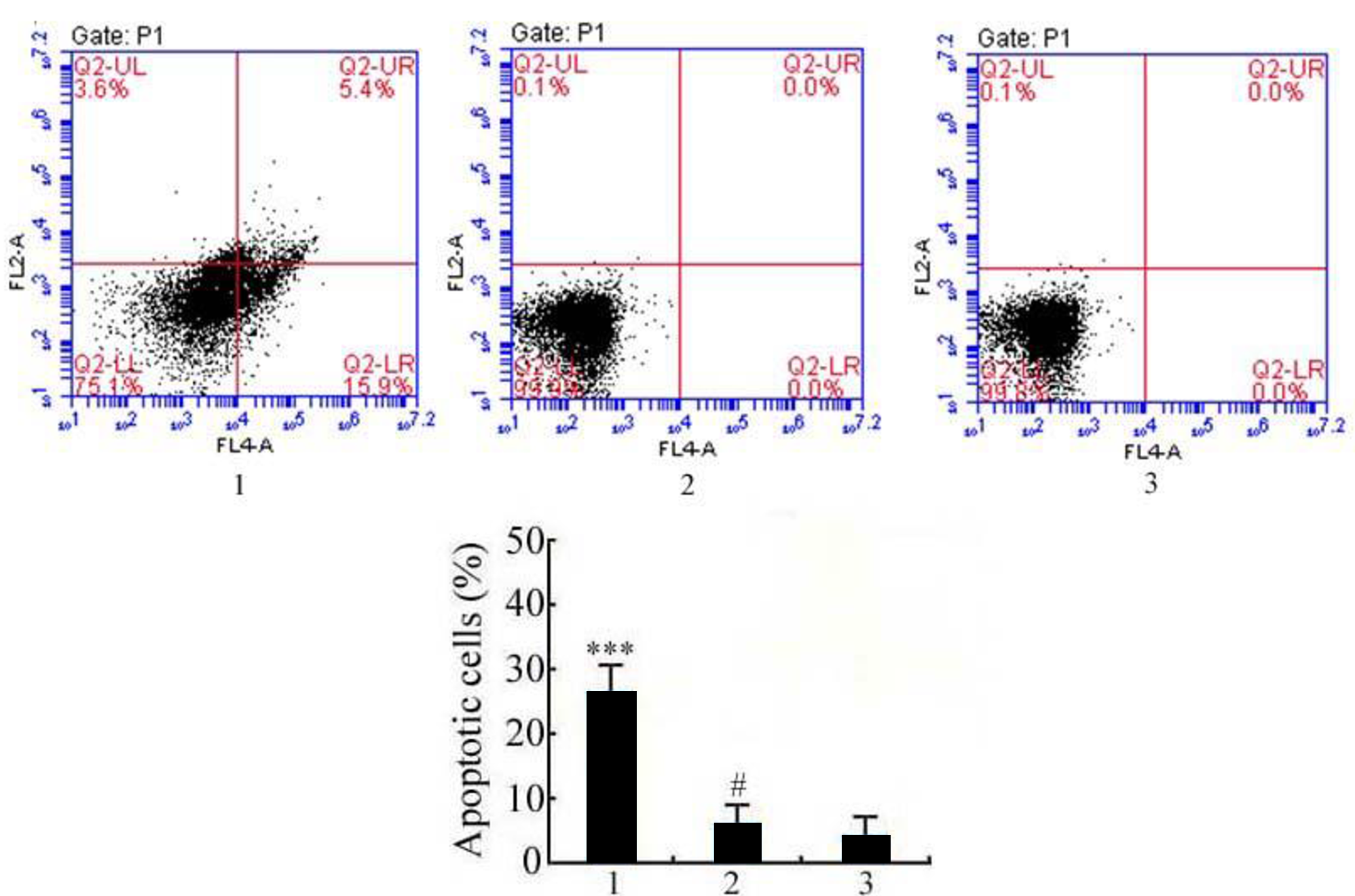

Supplement: Supplementary file 2 — Figure S2 HTR 8/SVneocells were treated with miR‐365 (group1), miR‐365 + anti‐ miR‐365 (group2), or empty vector (NC, group3). [file JCMM-21-2412-s002.tif]

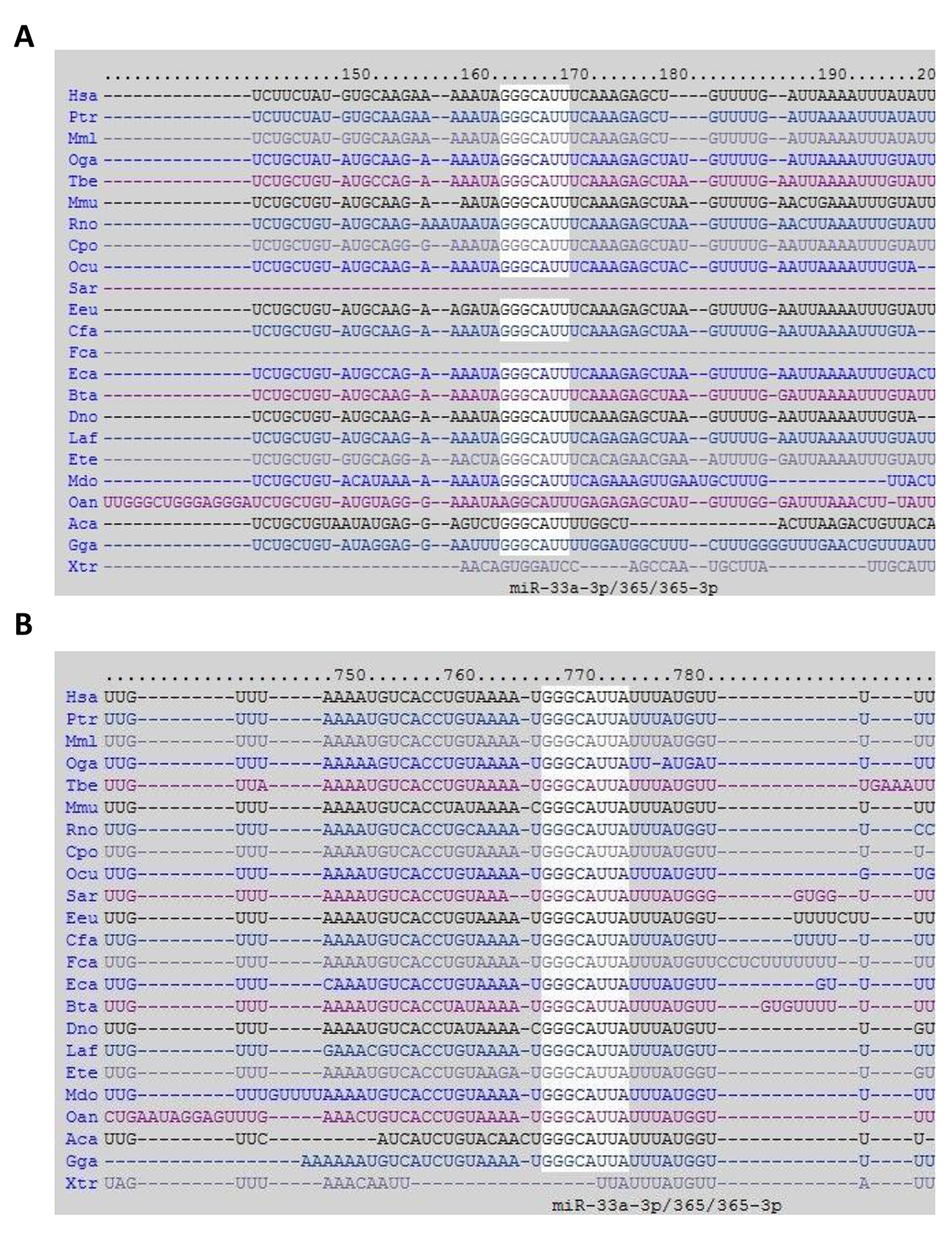

Supplement: Supplementary file 3 — Figure S3 The putative miR‐365 target sequences in the 3′ UTRs of SGK1 and SGK3 are highly conserved. [file JCMM-21-2412-s003.tif]

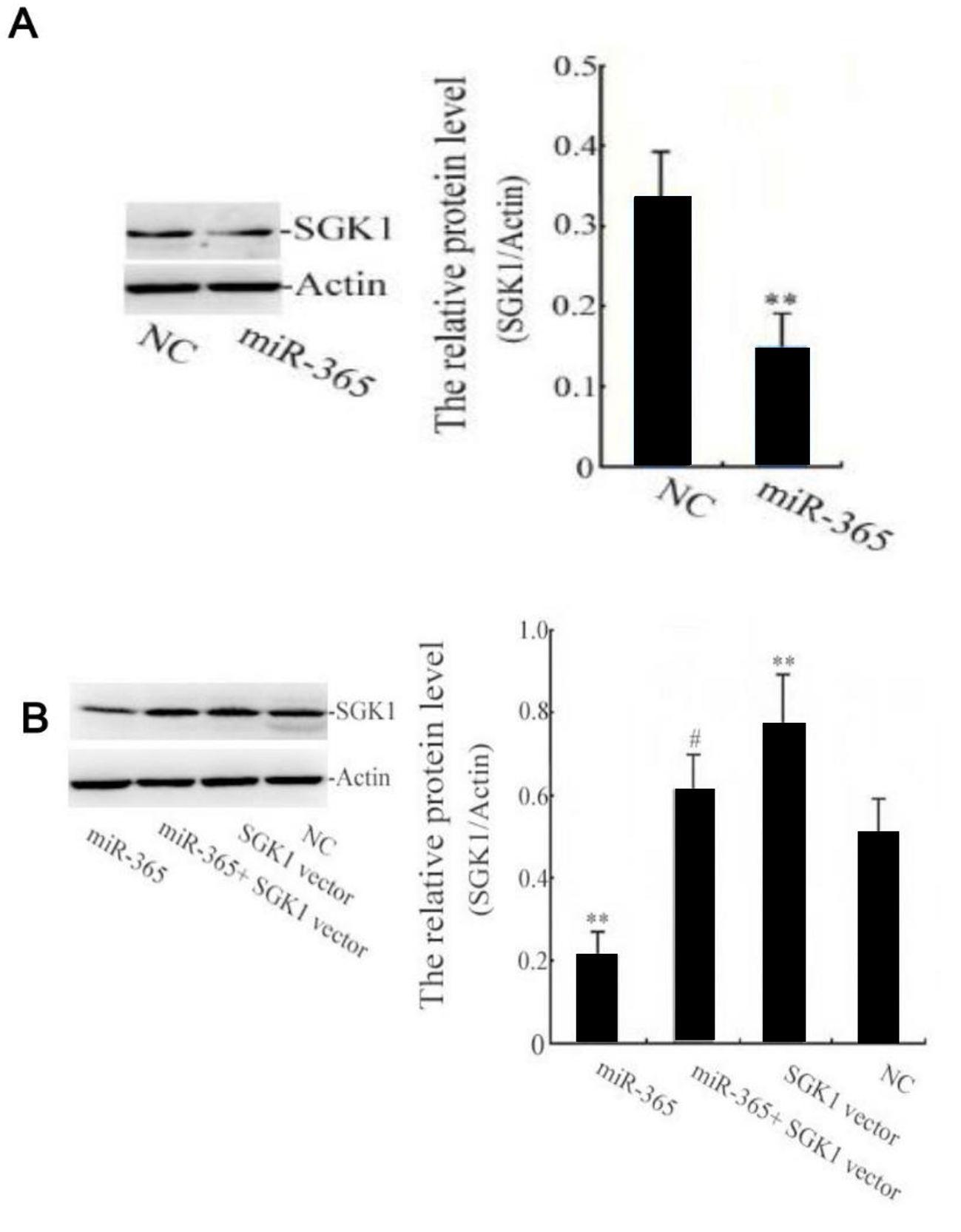

Supplement: Supplementary file 4 — Figure S4 SGK1 is a target of miR‐365. [file JCMM-21-2412-s004.tif]
