# Supplementary material for: Novel mechanism of miRNA‐365‐regulated trophoblast apoptosis in recurrent miscarriage
Source: J Cell Mol Med. 2017 Apr 10;21(10):2412–25. doi: 10.1111/jcmm.13163 (PMC5618703; doi:10.1111/jcmm.13163)
Supplement: Supplementary file 5 [file JCMM-21-2412-s005.pdf]

Supplementary Figure 1. Reduced MDM2 expression in RM. The relative expression level of MDM2 in decidual tissues of RM relative to IA was evaluated using Western blotting. Actin protein was tested as loading control. The data represent the means ± S.D. of three independent experiments (**, $p < 0.01$).

Supplementary Figure 2. HTR 8/SVneo cells were treated with miR-365 (group 1), miR-365+ anti-miR-365 (group 2), or empty vector (NC, group 3). Cells were stained with annexin V and analyzed by flow cytometry. The data represent means ± S.D. of three independent experiments (***, $p < 0.001$; #, $p > 0.05$ vs NC). Similar results were found in HPT-8 cells (data not shown).

Supplementary Figure 3. The putative miR-365 target sequences in the 3' UTRs of SGK1 and SGK3 are highly conserved. The evolutionary conservation of the GGGCAUUA sequence, which is complementary to the seed sequence of miR-365, is shown for SGK1 (panel **A**) and SGK3 (panel **B**) across species.

Supplementary Figure 4. SGK1 is a target of miR-365. (**A**) HTR 8/SVneo cells were treated with miR-365 mimic or empty vector (NC). After transfection, the protein expression of SGK1 was analyzed by Western blotting. Actin was tested as loading control. (**B**) HTR 8/SVneo cells were treated with miR-365, miR-365 + SGK1 vector, SGK1 vector or empty vector (NC). After transfection, the SGK1 expression levels were evaluated by Western blotting. SGK1 protein levels were normalized to actin protein levels. The data represent the means ± S.D. of three independent experiments (**, $p < 0.01$; #, $p > 0.05$ *vs* NC). Similar results were found in HPT-8 cells (data not shown).
